# 1,1′-(9-Octyl-9*H*-carbazole-3,6-di­yl)diethanone

**DOI:** 10.1107/S1600536810028928

**Published:** 2010-07-24

**Authors:** Aamer Saeed, Madiha Kazmi, Shahid Ameen Samra, Madiha Irfan, Michael Bolte

**Affiliations:** a Department of Chemistry, Quaid-i-Azam University, Islamabad 45320, Pakistan; b Institut für Anorganische Chemie, J. W. Goethe-Universität Frankfurt, Max-von-Laue-Strasse 7, 60438 Frankfurt/Main, Germany

## Abstract

The central structural element of the title compound, C_24_H_29_NO_2_, is a carbazole unit substituted with two acetyl residues and an octyl chain. The acetyl residues are nearly coplanar [dihedral angles = 5.37 (14) and 1.0 (3)°] with the carbazole unit which is essentially planar (r.m.s. deviation for all non-H atoms = 0.025 Å). The octyl chain adopts an all-*trans* conformation. The crystal packing is stabilized by C—H⋯O hydrogen bonds.

## Related literature

For details of the biological activity of carbazoles, see: Yamashita *et al.* (1992 ▶). For properties of aromatic carbazolyl groups, see: Law (1992 ▶). For the properties and applications of carbazole-containing polymers, see: Strohriegl & Grazulevicius (1997 ▶).
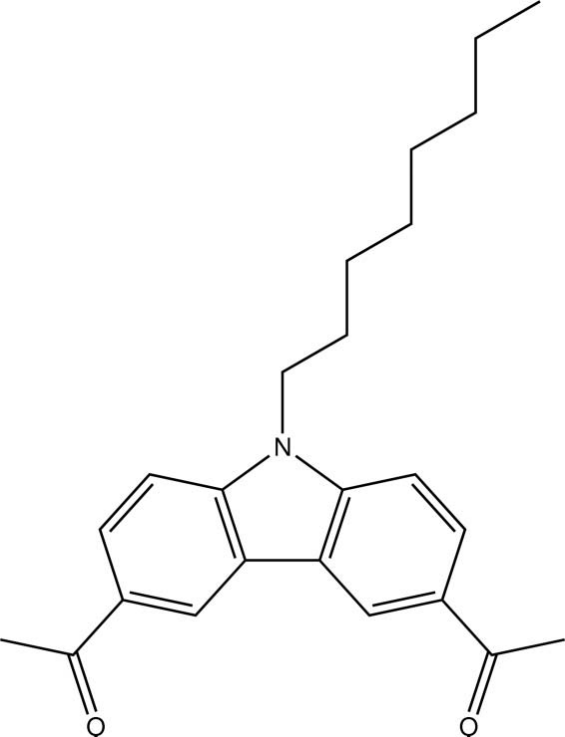


## Experimental

### 

#### Crystal data

C_24_H_29_NO_2_
*M* *_r_* = 363.48
Orthorhombic, 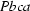

*a* = 18.746 (2) Å
*b* = 10.3842 (18) Å
*c* = 20.994 (3) Å
*V* = 4086.7 (10) Å^3^
*Z* = 8
Mo *K*α radiation
μ = 0.07 mm^−1^
*T* = 173 K
0.32 × 0.29 × 0.12 mm

#### Data collection

Stow IPDS II diffractometer
8614 measured reflections
3613 independent reflections
2024 reflections with *I* > 2σ(*I*)
*R*_int_ = 0.031

#### Refinement

*R*[*F* ^2^ > 2σ(*F* ^2^)] = 0.044
*wR*(*F* ^2^) = 0.086
*S* = 0.85
3613 reflections
248 parameters
H-atom parameters constrained
Δρ_max_ = 0.20 e Å^−3^
Δρ_min_ = −0.20 e Å^−3^

### 

Data collection: *X-AREA* (Stoe & Cie, 2001 ▶); cell refinement: *X-AREA*; data reduction: *X-AREA*; program(s) used to solve structure: *SHELXS97* (Sheldrick, 2008 ▶); program(s) used to refine structure: *SHELXL97* (Sheldrick, 2008 ▶); molecular graphics: *XP* (Sheldrick, 2008 ▶); software used to prepare material for publication: *SHELXL97*.

## Supplementary Material

Crystal structure: contains datablocks global, I. DOI: 10.1107/S1600536810028928/bv2151sup1.cif

Structure factors: contains datablocks I. DOI: 10.1107/S1600536810028928/bv2151Isup2.hkl

Additional supplementary materials:  crystallographic information; 3D view; checkCIF report

## Figures and Tables

**Table 1:** Hydrogen-bond geometry (Å, °)

*D*—H⋯*A*	*D*—H	H⋯*A*	*D*⋯*A*	*D*—H⋯*A*
C13—H13⋯O2^i^	0.95	2.59	3.474 (2)	154
C23—H23⋯O2^i^	0.95	2.39	3.298 (3)	160
C28—H28*A*⋯O1^i^	0.98	2.40	3.363 (2)	166
C26—H26⋯O1^ii^	0.95	2.54	3.484 (2)	173

Symmetry codes: (i) 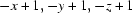; (ii) 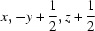.

**Fig. 1.**
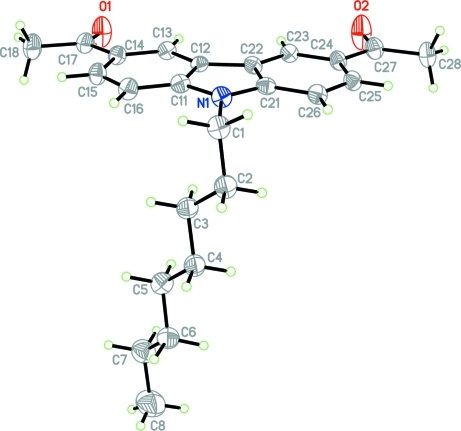
Molecular structure of title compound. Displacement ellipsoids are drawn at the 50% probability level.

## supplementary crystallographic information

### Comment

Carbazole and its derivatives have attracted extensive interest because of their biological activity (Yamashita *et al.* 1992). The carbazole based compounds demonstrate high thermal, morphological, chemical and environmental stability. Two basic properties of the fully aromatic carbazolyl group are the easy oxidizability of nitrogen atom and its ability to transport positive charge centers *via* the radical cation specie (Law, 1992). Carbazole containing polymers have been extensively studied for different applications due to their good hole transport and electroluminescent properties. (Strohriegl & Grazulevicius 1997). The title compound was prepared in order to study some photophysical properties of carbazole derivatives. It was synthesized by the reaction of carbazole and with octyl bromide in a two phase system of 50% aqueous KOH and benzene in the presence of tetrabutylammonium bromide as phase  transfer catalystfollowed by Friedel-Craft acetylation using anhydrous  aluminium chloride.

The central structural element of the title compound is a carbazole moiety substituted with two acetyl residues and an octyl-chain. The acetyl residues are coplanar [dihedral angles 5.37 (14)° and 1.0(3°] with the carbazole moiety which is essentially planar (r.m.s. deviation for all non-H atoms 0.025Å). The octyl chain adopts an all *trans* conformation. The crystal packing is stabilized by C—H···O hydrogen bonds.

### Experimental

Aluminium chloride, 4.0 g (3 mmol) and acetyl chloride, 2.35 g (3 mmol) were added successively to 10 ml of dry chloroform. The mixture was stirred for 10 minutes at 0 C to obtain a clear solution. A solution of 4.46 g (2 mmol) of N-octylcarbazole in 10 ml of dry chloroform was added drop wise to the above solution at 0°C during 15 minutes. The reaction mixture was stirred at room temperature for three hours. After the completion of the reaction (TLC control), the reaction mixture was poured into a stirred solution of 10% HCl (50 ml). The organic layer was separated, washed with distilled water three times and treated with anhydrous NaSO_4_. The solvent was removed *in vacuo* to leave a solid which was recrystallized from ethanol to afford title compund (85%) as dull green crystals having bread mold smell. m.p. 145 °C; Anal. calcd. for C_16_H_23_N_O_4: C, 65.51; H, 9.70; N, 4.77%; found: C, 65.58; H, 9.65; N, 4.81%.

### Refinement

H atoms could be located in a difference Fourier map. They were refined using a riding model with isotropic displacement parameters *U*_iso_(H) set to 1.2*U*_eq_(C) and with C—H ranging from 0.95Å to 0.99 or *U*_iso_(H) set to 1.5*U*_eq_(C_methyl_) and with C—H = 0.98 Å. The methyl groups were allowed to rotate but not to tip.

### Crystal data

**Table:**

C_24_H_29_NO_2_	*F*(000) = 1568
*M**_r_* = 363.48	*D*_x_ = 1.182 Mg m^−^^3^
Orthorhombic, *P**b**c**a*	Mo *K*α radiation, λ = 0.71073 Å
Hall symbol: -P 2ac 2ab	Cell parameters from 3794 reflections
*a* = 18.746 (2) Å	θ = 3.6–25.9°
*b* = 10.3842 (18) Å	µ = 0.07 mm^−^^1^
*c* = 20.994 (3) Å	*T* = 173 K
*V* = 4086.7 (10) Å^3^	Plate, colourless
*Z* = 8	0.32 × 0.29 × 0.12 mm

### Data collection

**Table:**

Stow IPDS II two-circle diffractometer	2024 reflections with *I* > 2σ(*I*)
Radiation source: fine-focus sealed tube	*R*_int_ = 0.031
graphite	θ_max_ = 25.0°, θ_min_ = 1.9°
ω scans	*h* = 0→22
8614 measured reflections	*k* = 0→12
3613 independent reflections	*l* = 0→24

### Refinement

**Table:**

Refinement on *F*^2^	Secondary atom site location: difference Fourier map
Least-squares matrix: full	Hydrogen site location: inferred from neighbouring sites
*R*[*F*^2^ > 2σ(*F*^2^)] = 0.044	H-atom parameters constrained
*wR*(*F*^2^) = 0.086	*w* = 1/[σ^2^(*F*_o_^2^) + (0.0389*P*)^2^] where *P* = (*F*_o_^2^ + 2*F*_c_^2^)/3
*S* = 0.85	(Δ/σ)_max_ = 0.001
3613 reflections	Δρ_max_ = 0.20 e Å^−^^3^
248 parameters	Δρ_min_ = −0.20 e Å^−^^3^
0 restraints	Extinction correction: *SHELXL97* (Sheldrick, 2008), Fc^*^=kFc[1+0.001xFc^2^λ^3^/sin(2θ)]^-1/4^
Primary atom site location: structure-invariant direct methods	Extinction coefficient: 0.0019 (3)

### Special details

**Table:**

Geometry. All e.s.d.'s (except the e.s.d. in the dihedral angle between two l.s. planes) are estimated using the full covariance matrix. The cell e.s.d.'s are taken into account individually in the estimation of e.s.d.'s in distances, angles and torsion angles; correlations between e.s.d.'s in cell parameters are only used when they are defined by crystal symmetry. An approximate (isotropic) treatment of cell e.s.d.'s is used for estimating e.s.d.'s involving l.s. planes.
Refinement. Refinement of *F*^2^ against ALL reflections. The weighted *R*-factor *wR* and goodness of fit *S* are based on *F*^2^, conventional *R*-factors *R* are based on *F*, with *F* set to zero for negative *F*^2^. The threshold expression of *F*^2^ > σ(*F*^2^) is used only for calculating *R*-factors(gt) *etc*. and is not relevant to the choice of reflections for refinement. *R*-factors based on *F*^2^ are statistically about twice as large as those based on *F*, and *R*- factors based on ALL data will be even larger.

### Fractional atomic coordinates and isotropic or equivalent isotropic displacement parameters (Å^2^)

**Table:**

	*x*	*y*	*z*	*U*_iso_*/*U*_eq_	
N1	0.70663 (9)	0.23596 (8)	0.66696 (8)	0.0259 (4)	
O1	0.68112 (9)	0.13339 (7)	0.37076 (7)	0.0482 (4)	
O2	0.45645 (10)	0.58614 (10)	0.56767 (8)	0.0579 (5)	
C1	0.75428 (11)	0.21735 (9)	0.72139 (9)	0.0300 (5)	
H1A	0.7787	0.1332	0.7170	0.036*	
H1B	0.7255	0.2147	0.7609	0.036*	
C2	0.81004 (11)	0.32270 (10)	0.72736 (9)	0.0340 (5)	
H2A	0.8416	0.3023	0.7638	0.041*	
H2B	0.7856	0.4050	0.7370	0.041*	
C3	0.85589 (12)	0.34086 (10)	0.66809 (10)	0.0335 (5)	
H3A	0.8247	0.3640	0.6318	0.040*	
H3B	0.8797	0.2584	0.6577	0.040*	
C4	0.91269 (11)	0.44553 (10)	0.67644 (10)	0.0342 (5)	
H4A	0.8892	0.5259	0.6907	0.041*	
H4B	0.9463	0.4186	0.7103	0.041*	
C5	0.95472 (12)	0.47302 (9)	0.61566 (10)	0.0358 (5)	
H5A	0.9770	0.3921	0.6007	0.043*	
H5B	0.9213	0.5024	0.5822	0.043*	
C6	1.01263 (12)	0.57443 (10)	0.62449 (10)	0.0376 (5)	
H6A	1.0499	0.5396	0.6531	0.045*	
H6B	0.9916	0.6509	0.6455	0.045*	
C7	1.04725 (13)	0.61615 (10)	0.56216 (12)	0.0472 (6)	
H7A	1.0098	0.6487	0.5331	0.057*	
H7B	1.0695	0.5402	0.5418	0.057*	
C8	1.10405 (14)	0.72094 (10)	0.57099 (14)	0.0696 (9)	
H8A	1.0812	0.8006	0.5855	0.104*	
H8B	1.1282	0.7366	0.5303	0.104*	
H8C	1.1390	0.6926	0.6028	0.104*	
C11	0.71650 (11)	0.18471 (9)	0.60607 (9)	0.0251 (4)	
C12	0.66565 (10)	0.23960 (8)	0.56417 (9)	0.0217 (4)	
C13	0.66665 (11)	0.20425 (9)	0.49974 (9)	0.0249 (4)	
H13	0.6334	0.2406	0.4707	0.030*	
C14	0.71716 (11)	0.11499 (9)	0.47877 (10)	0.0260 (4)	
C15	0.76638 (11)	0.06208 (9)	0.52180 (10)	0.0295 (5)	
H15	0.8003	0.0013	0.5068	0.035*	
C16	0.76684 (11)	0.09608 (9)	0.58571 (9)	0.0290 (5)	
H16	0.8004	0.0599	0.6145	0.035*	
C17	0.72007 (12)	0.08031 (9)	0.40940 (10)	0.0310 (5)	
C18	0.77287 (12)	−0.01784 (9)	0.38732 (9)	0.0405 (6)	
H18A	0.7674	−0.0314	0.3414	0.061*	
H18B	0.7646	−0.0992	0.4098	0.061*	
H18C	0.8213	0.0127	0.3963	0.061*	
C21	0.65132 (10)	0.32388 (11)	0.66472 (9)	0.0240 (4)	
C22	0.62341 (11)	0.32930 (10)	0.60171 (9)	0.0234 (4)	
C23	0.56768 (10)	0.41338 (8)	0.58793 (9)	0.0249 (4)	
H23	0.5486	0.4182	0.5461	0.030*	
C24	0.54038 (10)	0.49026 (10)	0.63647 (9)	0.0249 (4)	
C25	0.56898 (11)	0.48308 (11)	0.69854 (9)	0.0264 (4)	
H25	0.5495	0.5364	0.7309	0.032*	
C26	0.62438 (11)	0.40090 (8)	0.71366 (9)	0.0262 (4)	
H26	0.6434	0.3969	0.7556	0.031*	
C27	0.48115 (12)	0.58090 (11)	0.62149 (10)	0.0320 (5)	
C28	0.44961 (11)	0.66343 (9)	0.67328 (10)	0.0367 (5)	
H28A	0.4168	0.7263	0.6544	0.055*	
H28B	0.4879	0.7088	0.6957	0.055*	
H28C	0.4235	0.6089	0.7034	0.055*	

### Atomic displacement parameters (Å^2^)

**Table:**

	*U*^11^	*U*^22^	*U*^33^	*U*^12^	*U*^13^	*U*^23^
N1	0.0267 (9)	0.0301 (4)	0.0209 (9)	0.0028 (7)	−0.0033 (8)	0.0032 (7)
O1	0.0590 (11)	0.0599 (4)	0.0255 (9)	0.0280 (8)	−0.0093 (9)	−0.0055 (6)
O2	0.0641 (12)	0.0820 (6)	0.0276 (9)	0.0404 (9)	−0.0134 (9)	−0.0098 (9)
C1	0.0336 (12)	0.0367 (4)	0.0198 (11)	0.0043 (9)	−0.0051 (11)	0.0048 (8)
C2	0.0343 (13)	0.0419 (4)	0.0258 (12)	0.0033 (10)	−0.0061 (10)	−0.0030 (9)
C3	0.0323 (11)	0.0375 (4)	0.0306 (12)	0.0017 (9)	−0.0050 (11)	−0.0043 (9)
C4	0.0329 (11)	0.0383 (5)	0.0315 (13)	0.0030 (9)	−0.0040 (11)	−0.0048 (9)
C5	0.0390 (13)	0.0353 (4)	0.0330 (13)	0.0008 (9)	−0.0035 (11)	0.0015 (7)
C6	0.0354 (12)	0.0404 (4)	0.0372 (13)	0.0002 (10)	−0.0037 (11)	−0.0020 (8)
C7	0.0437 (15)	0.0484 (5)	0.0494 (16)	−0.0020 (10)	0.0038 (13)	0.0011 (10)
C8	0.0539 (19)	0.0634 (6)	0.092 (2)	−0.0126 (11)	0.0141 (18)	0.0065 (11)
C11	0.0276 (11)	0.0257 (4)	0.0219 (11)	−0.0021 (9)	0.0010 (10)	0.0025 (8)
C12	0.0219 (10)	0.0199 (4)	0.0233 (11)	−0.0022 (8)	−0.0003 (9)	0.0009 (7)
C13	0.0269 (10)	0.0267 (4)	0.0211 (11)	0.0002 (8)	−0.0018 (10)	0.0013 (7)
C14	0.0292 (10)	0.0264 (4)	0.0224 (11)	0.0011 (8)	−0.0007 (10)	−0.0010 (8)
C15	0.0316 (12)	0.0272 (4)	0.0298 (12)	0.0070 (8)	−0.0010 (10)	−0.0001 (8)
C16	0.0315 (13)	0.0287 (4)	0.0268 (11)	0.0059 (9)	−0.0055 (10)	0.0016 (8)
C17	0.0344 (12)	0.0304 (4)	0.0281 (12)	0.0030 (9)	−0.0002 (11)	−0.0031 (8)
C18	0.0476 (15)	0.0435 (5)	0.0303 (13)	0.0130 (10)	−0.0009 (11)	−0.0073 (7)
C21	0.0238 (10)	0.0266 (4)	0.0217 (11)	−0.0039 (8)	0.0013 (9)	0.0037 (8)
C22	0.0228 (10)	0.0275 (4)	0.0200 (10)	−0.0014 (9)	0.0015 (9)	0.0026 (8)
C23	0.0252 (10)	0.0293 (4)	0.0202 (10)	0.0004 (8)	−0.0014 (9)	0.0009 (7)
C24	0.0243 (10)	0.0305 (5)	0.0200 (11)	0.0034 (9)	0.0012 (10)	0.0012 (8)
C25	0.0273 (10)	0.0315 (4)	0.0204 (11)	0.0002 (9)	0.0038 (9)	−0.0006 (8)
C26	0.0281 (11)	0.0324 (4)	0.0180 (10)	−0.0020 (8)	0.0014 (10)	0.0013 (7)
C27	0.0325 (13)	0.0384 (5)	0.0251 (12)	0.0054 (10)	0.0012 (10)	0.0012 (9)
C28	0.0377 (14)	0.0439 (4)	0.0285 (12)	0.0140 (9)	0.0032 (11)	−0.0022 (8)

### Geometric parameters (Å, °)

**Table:**

N1—C21	1.382 (2)	C11—C16	1.386 (2)
N1—C11	1.397 (2)	C11—C12	1.417 (2)
N1—C1	1.463 (2)	C12—C13	1.402 (2)
O1—C17	1.223 (2)	C12—C22	1.455 (2)
O2—C27	1.222 (3)	C13—C14	1.396 (2)
C1—C2	1.518 (2)	C13—H13	0.9500
C1—H1A	0.9900	C14—C15	1.403 (3)
C1—H1B	0.9900	C14—C17	1.501 (3)
C2—C3	1.524 (3)	C15—C16	1.388 (3)
C2—H2A	0.9900	C15—H15	0.9500
C2—H2B	0.9900	C16—H16	0.9500
C3—C4	1.532 (2)	C17—C18	1.494 (2)
C3—H3A	0.9900	C18—H18A	0.9800
C3—H3B	0.9900	C18—H18B	0.9800
C4—C5	1.527 (3)	C18—H18C	0.9800
C4—H4A	0.9900	C21—C26	1.397 (2)
C4—H4B	0.9900	C21—C22	1.424 (3)
C5—C6	1.524 (2)	C22—C23	1.392 (2)
C5—H5A	0.9900	C23—C24	1.392 (2)
C5—H5B	0.9900	C23—H23	0.9500
C6—C7	1.524 (3)	C24—C25	1.411 (3)
C6—H6A	0.9900	C24—C27	1.489 (2)
C6—H6B	0.9900	C25—C26	1.381 (2)
C7—C8	1.534 (3)	C25—H25	0.9500
C7—H7A	0.9900	C26—H26	0.9500
C7—H7B	0.9900	C27—C28	1.505 (3)
C8—H8A	0.9800	C28—H28A	0.9800
C8—H8B	0.9800	C28—H28B	0.9800
C8—H8C	0.9800	C28—H28C	0.9800
			
C21—N1—C11	108.65 (15)	N1—C11—C12	109.01 (13)
C21—N1—C1	124.87 (15)	C13—C12—C11	119.00 (14)
C11—N1—C1	125.69 (15)	C13—C12—C22	134.26 (16)
N1—C1—C2	112.93 (12)	C11—C12—C22	106.70 (16)
N1—C1—H1A	109.0	C14—C13—C12	119.16 (17)
C2—C1—H1A	109.0	C14—C13—H13	120.4
N1—C1—H1B	109.0	C12—C13—H13	120.4
C2—C1—H1B	109.0	C13—C14—C15	120.19 (17)
H1A—C1—H1B	107.8	C13—C14—C17	119.33 (17)
C1—C2—C3	114.21 (15)	C15—C14—C17	120.44 (15)
C1—C2—H2A	108.7	C16—C15—C14	121.80 (15)
C3—C2—H2A	108.7	C16—C15—H15	119.1
C1—C2—H2B	108.7	C14—C15—H15	119.1
C3—C2—H2B	108.7	C11—C16—C15	117.59 (17)
H2A—C2—H2B	107.6	C11—C16—H16	121.2
C2—C3—C4	112.73 (16)	C15—C16—H16	121.2
C2—C3—H3A	109.0	O1—C17—C18	119.81 (17)
C4—C3—H3A	109.0	O1—C17—C14	120.92 (15)
C2—C3—H3B	109.0	C18—C17—C14	119.25 (17)
C4—C3—H3B	109.0	C17—C18—H18A	109.5
H3A—C3—H3B	107.8	C17—C18—H18B	109.5
C5—C4—C3	113.32 (16)	H18A—C18—H18B	109.5
C5—C4—H4A	108.9	C17—C18—H18C	109.5
C3—C4—H4A	108.9	H18A—C18—H18C	109.5
C5—C4—H4B	108.9	H18B—C18—H18C	109.5
C3—C4—H4B	108.9	N1—C21—C26	128.64 (17)
H4A—C4—H4B	107.7	N1—C21—C22	109.47 (16)
C6—C5—C4	113.28 (17)	C26—C21—C22	121.87 (16)
C6—C5—H5A	108.9	C23—C22—C21	119.59 (16)
C4—C5—H5A	108.9	C23—C22—C12	134.23 (17)
C6—C5—H5B	108.9	C21—C22—C12	106.15 (15)
C4—C5—H5B	108.9	C24—C23—C22	118.92 (17)
H5A—C5—H5B	107.7	C24—C23—H23	120.5
C5—C6—C7	113.29 (18)	C22—C23—H23	120.5
C5—C6—H6A	108.9	C23—C24—C25	120.40 (16)
C7—C6—H6A	108.9	C23—C24—C27	118.81 (17)
C5—C6—H6B	108.9	C25—C24—C27	120.78 (17)
C7—C6—H6B	108.9	C26—C25—C24	122.04 (16)
H6A—C6—H6B	107.7	C26—C25—H25	119.0
C6—C7—C8	113.2 (2)	C24—C25—H25	119.0
C6—C7—H7A	108.9	C25—C26—C21	117.18 (17)
C8—C7—H7A	108.9	C25—C26—H26	121.4
C6—C7—H7B	108.9	C21—C26—H26	121.4
C8—C7—H7B	108.9	O2—C27—C24	120.39 (18)
H7A—C7—H7B	107.8	O2—C27—C28	119.58 (18)
C7—C8—H8A	109.5	C24—C27—C28	120.00 (18)
C7—C8—H8B	109.5	C27—C28—H28A	109.5
H8A—C8—H8B	109.5	C27—C28—H28B	109.5
C7—C8—H8C	109.5	H28A—C28—H28B	109.5
H8A—C8—H8C	109.5	C27—C28—H28C	109.5
H8B—C8—H8C	109.5	H28A—C28—H28C	109.5
C16—C11—N1	128.72 (17)	H28B—C28—H28C	109.5
C16—C11—C12	122.26 (16)		
			
C21—N1—C1—C2	−76.3 (2)	C13—C14—C17—C18	−178.38 (14)
C11—N1—C1—C2	92.42 (16)	C15—C14—C17—C18	4.0 (2)
N1—C1—C2—C3	−56.0 (2)	C11—N1—C21—C26	−177.94 (14)
C1—C2—C3—C4	−178.57 (13)	C1—N1—C21—C26	−7.6 (2)
C2—C3—C4—C5	−174.96 (14)	C11—N1—C21—C22	0.98 (17)
C3—C4—C5—C6	−178.36 (13)	C1—N1—C21—C22	171.30 (15)
C4—C5—C6—C7	−171.59 (14)	N1—C21—C22—C23	−179.02 (12)
C5—C6—C7—C8	178.39 (15)	C26—C21—C22—C23	0.0 (2)
C21—N1—C11—C16	178.24 (14)	N1—C21—C22—C12	−0.66 (17)
C1—N1—C11—C16	8.0 (2)	C26—C21—C22—C12	178.35 (13)
C21—N1—C11—C12	−0.92 (16)	C13—C12—C22—C23	0.5 (3)
C1—N1—C11—C12	−171.13 (13)	C11—C12—C22—C23	178.11 (17)
C16—C11—C12—C13	−0.7 (2)	C13—C12—C22—C21	−177.50 (17)
N1—C11—C12—C13	178.53 (14)	C11—C12—C22—C21	0.10 (16)
C16—C11—C12—C22	−178.73 (13)	C21—C22—C23—C24	−0.2 (2)
N1—C11—C12—C22	0.49 (16)	C12—C22—C23—C24	−177.96 (16)
C11—C12—C13—C14	0.7 (2)	C22—C23—C24—C25	0.2 (2)
C22—C12—C13—C14	178.06 (14)	C22—C23—C24—C27	179.51 (15)
C12—C13—C14—C15	−0.3 (2)	C23—C24—C25—C26	0.0 (2)
C12—C13—C14—C17	−177.90 (14)	C27—C24—C25—C26	−179.33 (13)
C13—C14—C15—C16	−0.2 (2)	C24—C25—C26—C21	−0.2 (2)
C17—C14—C15—C16	177.43 (16)	N1—C21—C26—C25	178.98 (14)
N1—C11—C16—C15	−178.79 (15)	C22—C21—C26—C25	0.2 (2)
C12—C11—C16—C15	0.3 (2)	C23—C24—C27—O2	1.0 (3)
C14—C15—C16—C11	0.2 (2)	C25—C24—C27—O2	−179.67 (18)
C13—C14—C17—O1	3.4 (2)	C23—C24—C27—C28	178.84 (14)
C15—C14—C17—O1	−174.24 (15)	C25—C24—C27—C28	−1.8 (2)

### Hydrogen-bond geometry (Å, °)

**Table:**

*D*—H···*A*	*D*—H	H···*A*	*D*···*A*	*D*—H···*A*
C13—H13···O2^i^	0.95	2.59	3.474 (2)	154
C23—H23···O2^i^	0.95	2.39	3.298 (3)	160
C28—H28A···O1^i^	0.98	2.40	3.363 (2)	166
C26—H26···O1^ii^	0.95	2.54	3.484 (2)	173

Symmetry codes: (i) −*x*+1, −*y*+1, −*z*+1; (ii) *x*, −*y*+1/2, *z*+1/2.
